# Frugivore-Mediated Selection in A Habitat Transformation Scenario

**DOI:** 10.1038/srep45371

**Published:** 2017-03-28

**Authors:** Francisco E. Fontúrbel, Rodrigo Medel

**Affiliations:** 1 Instituto de Biología, Facultad de Ciencias, Pontificia Universidad Católica de Valparaíso. Universidad 330, Curauma 2340000, Valparaíso, Chile; 2 Departamento de Ciencias Ecológicas, Facultad de Ciencias, Universidad de Chile. Las Palmeras 3425, Ñuñoa 7800024, Santiago, Chile

## Abstract

Plant-animal interactions are strong drivers of phenotypic evolution. However, the extent to which anthropogenic habitat transformation creates new selective scenarios for plant-animal interactions is a little explored subject. We examined the effects of native forest replacement by exotic *Eucalyptus* trees on the frugivore-mediated phenotypic selection coefficients imposed by the relict marsupial *Dromiciops gliroides* upon traits involved in frugivore attraction and germination success of the mistletoe *Tristerix corymbosus* (Loranthaceae). We found significant gradients for seed weight and sugar content along the native - transformed habitat gradient. While selection for larger seed weight was more relevant in native habitats, fruits with intermediate sugar content were promoted in transformed habitats. The spatial habitat structure and microclimate features such as the degree of sunlight received influenced the natural selection processes, as they correlated with the phenotypic traits analysed. The response of this plant-frugivore interaction to human disturbance seemed to be context-dependent, in which extremely transformed habitats would offer new opportunities for natural selection on dispersal-related traits. Even in recent transformation events like this, human disturbance acts as a strong contemporary evolution driver.

Plant-animal interactions are strong drivers of reciprocal phenotypic evolution^1^, leading to coevolved traits that are dynamic through time and space^2^,^3^. However, this complex eco-evolutionary system is challenged by a world in constant change in which many anthropogenic biodiversity-loss drivers are altering natural habitats^4^. Each species responds to habitat disturbance according to their life history traits^5^, which explains, at least in part, the variety of responses to human disturbance observed in nature^6^,^7^. For example, while habitat fragmentation often modifies species abundances due to area and edge effects^8^,^9^, habitat transformation, involving the partial or complete replacement of the native vegetation by exotic species^10^,^11^, usually leads to more dramatic effects such as local extinction, arrival of new species, and changes in species composition.

Even though seed dispersal is a key stage in a plant’s life cycle that determines the demographic response to human-induced habitat disturbance^12^,^13^, studies examining phenotypic selection on seed dispersal in human-induced transformed habitats are scarce compared to those focused on pollination interactions (e.g., ref. 14). Seed dispersal shapes spatial and genetic structure of plants^15^ which determines the potential of plant populations to respond to changes in selective scenarios. Recent studies showed that anthropogenic defaunation collapses seed dispersal and changes allelic frequencies^16^,^17^, but little is known about potential contemporary evolution on seed dispersal^18^. Human activities are remarkably strong selection forces^19^ which are able to change the eco-evolutionary scenario in which seed dispersal interactions occur. We examined the importance of frugivore-mediated phenotypic selection in a highly specialized system (a mistletoe with a single disperser species), which would allow us to examine the effects of frugivore-mediated selection without the background noise that redundant frugivore species might impose on generalist plant-frugivore system, along a habitat disturbance gradient to answer the following questions: (1) Does frugivore-mediated selection on fruit traits change along a habitat transformation gradient? (2) Do native and transformed habitats differ sufficiently in structural and microclimate conditions to create spatially variable selection scenarios for fruit traits? We hypothesize that habitat structural and microclimate features affect dispersal-related fruit traits and consequently habitat transformation would change the magnitude, direction and significance of phenotypic selection coefficients acting upon them.

## Results

**Figure 1:**
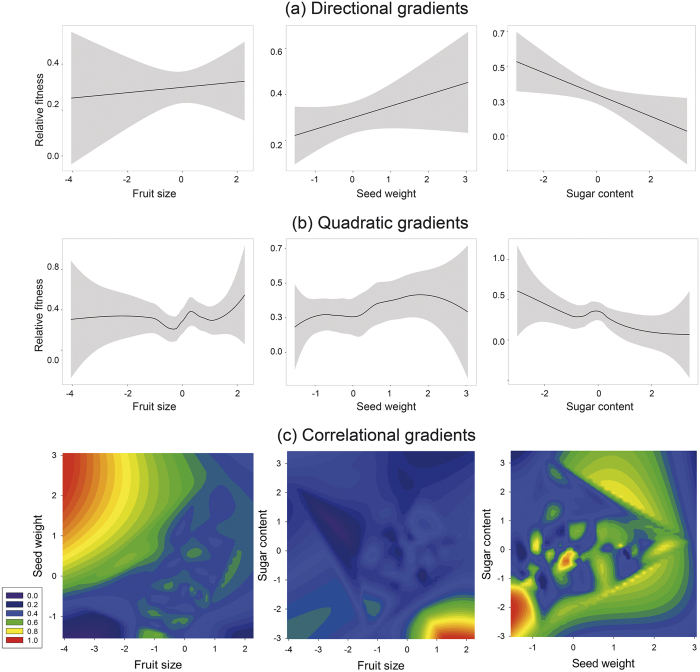
Frugivore-mediated selection gradients estimated for *Tristerix corymbosus* fruit traits. (**a**) Directional selection gradients, lines depict the fit of generalized additive models for each trait; (**b**) quadratic selection gradients, lines depict cubic splines (based on loess); (**c**) correlational gradients, surfaces depict pairwise trait combinations with relative fitness showed in a colour scale.

Crop size was correlated with most of the plant traits and fecundity components, whereas inter-trait correlation was significant only for fruit diameter and seed weight (Table S1, available online as Supplementary Information). Also seed weight was positively correlated to germination. The proportion of native habitat was negatively correlated to seed weight and fruit removal at 50–100 m and 100–250 m scales, and no variable was correlated at 0–50 m habitat scale (Table S1). Seed weight presented a positive significant directional gradient, whereas sugar content had a negative significant gradient (Fig. 1a); fruit diameter has no significant gradient (Table 1). Sample size should be increased up to 6,232 plants to detect a significant directional gradient for fruit diameter (far exceeding the number of plants available at the study area). We detected a disruptive gradient for seed weight and a stabilizing gradient for sugar content (Table 1; Fig. 1b). We found a significant correlational gradient for fruit diameter and seed weight (Fig. 1c), the remaining correlational gradients were non-significant (Table 1). Spatial structure was significant (P < 0.001) in both linear and non-linear selection gradient analyses, but crop size covariate was not significant in any case.

**Figure 2:**
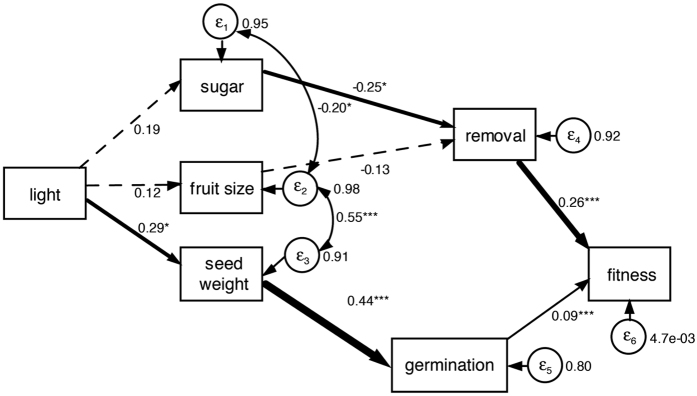
SEM diagram of the best-fitted model. Dashed arrows represent non-significant effects; line width is proportional to the path coefficient values. Significance of path coefficients: *P < 0.05, ***P < 0.001.

From the six SEM models fitted, we kept the model with the best fit (χ^2^_11_ = 12.75, P = 0.31; detailed SEM results are presented in Table S2). Such model shows that light had a positive influence on seed weight, which had a strong positive effect on seed germination, and then seed germination also had a positive effect on plant fitness (although not that strong as the effect of seed weight on germination). On the other hand, light had a positive but non-significant effect on both fruit size and sugar content, as those plant traits are negatively correlated (i.e., larger fruits have less sugar content). Then, sugar content had a negative influence on fruit removal, which has a strong positive effect on plant fitness. Additionally, fruit size positively covaried with seed weight (Fig. 2). Crop size was negatively related to relative plant fitness (Figure S1) and was strongly affected by the spatial structure of plants. Plant fitness reached its maximum at those plants with small crop sizes that usually have most of their fruits removed at the end of the summer. As crop size increases, a greater proportion of the available fruits remains on the plant, decreasing the relative plant fitness due to a reduction of its quantitative component.

For comparative purposes, we separated sampled plants into two groups, aiming to contrast plant traits and fecundity components between native and transformed types, being only fruit removal and relative plant fitness significantly different between habitats (Table S3); the global comparison showed no between-habitat differences (non-parametric MANOVA F_1,69_ = 0.88, P = 0.397). According to the ANCOVA results, habitat has an important effect (although marginal after sequential Bonferroni adjustment, P = 0.058) in explaining plant fitness differences along the transformation gradient; sugar content showed to be relevant to explain those differences in plant fitness (Table 2).

Fruit diameter does not vary between experimentally shaded and gap mistletoes in the field experiment (paired t = 0.859, P = 0.402). Conversely, seed dry weight (paired t = −3.071, P = 0.007) and sugar content (paired t = −4.198, P < 0.001) were significantly larger at those mistletoes experimentally exposed to sunlight. Seed weight and sugar content were positively correlated to luminosity, also sugar content was negatively correlated to shrub cover (Table S4). Those correlations suggest that less shaded plants tend to produce sweeter fruits.

**Figure 3:**
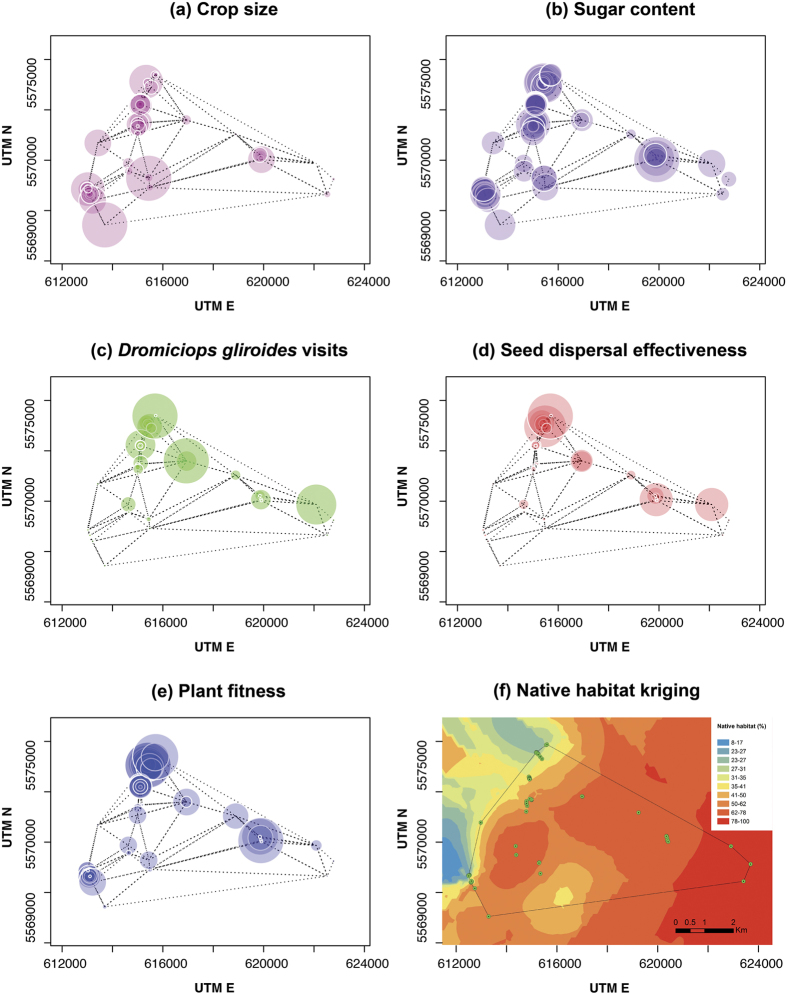
Spatially explicit representation of the sampled mistletoes, superimposing data for: (**a**) *Tristerix corymbosus* crop size, (**b**) fruit sugar content, (**c**) *Dromiciops gliroides* visits, (**d**) Seed disperser effectiveness, and (**e**) Plant’s relative fitness; bubble size is proportional to the magnitude of each variable. Delaunay triangulations in panels (**a** to **e**) are showed in dashed lines. Panel (**f**) shows a Kriging map of the proportion of native habitat, warmer colours indicate more abundant native cover (Kriging specifications: type = ordinary, transformation = arcsin, number of lags = 12, lag size = 401.39, nugget = 0.03, partial sill = 0.06).

As spatial structure was significant at all spatially explicit GAM models that we fitted, we used a graphical approach to visually contrast the observed *T. corymbosus* fecundity patterns with plants’ arrangement (Fig. 3). Crop size was larger at those areas dominated by transformed habitat (Fig. 3a), whereas fruit sugar content was variable along the habitat transformation gradient (Fig. 3b). *Dromiciops gliroides* visits were more frequent at those sites where *T. corymbosus* plants were densely aggregated (Fig. 3c). Seed disperser effectiveness (Fig. 3d) showed a similar trend, but *T. corymbosus* aggregations located at sites with intermediate native habitat cover (50–60%, confront with Fig. 3f) had lower disperser effectiveness despite having been visited many times by *D. gliroides*. A similar pattern is observed for the resulting plant fitness (Fig. 3e), but results suggest conflicting selection forces imposed from the qualitative component (i.e., seed germination) that balances the fitness of plants along the landscape. On the one hand, those sites with dense plant aggregations, large visit rates and higher effectiveness values had slightly lower plant fitness values due to reduced germination rates. On the other hand, sites that received few visits and presented low effectiveness values had slightly larger plant fitness values due to a higher seed germination rate (confront panels 3c–3e). Finally, comparing panels, those results with the abundance of native habitat at the landscape, it is evident that dense plant aggregations and larger relative plant fitness values occur at those sites dominated whether by transformed or by native habitat which suggests that frugivory and seed dispersal performed best at either extreme situation but have a lower performance at intermediate situations.

## Discussion

This highly specialized mistletoe-disperser system has persisted at transformed habitats. It seems to be benefited by sunlight exposure that positively affects seed size and germination. Studies conducted in fragmented landscapes showed that plant-frugivore interactions are resilient and could persist in fragmented habitats^20^,^21^; however, those studies have not evaluated the impact of habitat loss on selective forces. Our results show that habitat transformation promotes frugivore-mediated selection on some fruit traits, creating a novel selective scenario compared to the non-disturbed condition (i.e., the native forest). We found that fruits with larger seeds had greater germination rates, which is expected since large seeds have large energy reserves and are capable to maintain the embryo especially in harsh environmental conditions^22^. In this sense, as transformed habitat become dominant, seeds were larger (probably due to sunlight exposure) and were also removed in a greater extent, stressing the role of the large seeds as a germination success factor. This situation suggests that conflicting selection forces may act upon this mutualistic system, as for the seed disperser vector consuming fruits with larger seeds is more costly than rewarding, but this largely favours germination chances^23^. Despite seed size played a decisive role to plant recruitment in disturbed habitats, fruit size had no effect on any situation, concurring to what was found in *Prunus mahaleb* in Spain^12^. This outcome may result from the positive correlation between fruit size and seed weight, being fruit size indirectly selected through seed size^23^. In other frugivore-mediated selection studies, fruit size presented positive and significant selection gradients whereas seed size does not^24^,^25^, suggesting that selection on single-seeded fruits could be targeted on fruit or seed size as both plant traits are positively correlated. In our results, fruit size showed significant covariances with seed weight (positive) and sugar content (negative) according to our SEM model that agrees with the correlational selection gradients here reported. Larger seeds imply larger fruits, which typically have less sugar content as result of sugar dilution in a greater pulp volume. Crop size was negatively correlated to fruit removal and consequently to seed disperser effectiveness, as found for *P. mahaleb*^12^. Mistletoes with less than 30 fruits usually had no remaining fruits left at the end of the austral summer (March-April), whereas those plants with large crop sizes (>100) had a large proportion of their fruits undispersed. Nevertheless, if we consider the absolute number of seeds dispersed instead of a removal proportion, plants with large crop sizes may have more seeds removed but they would experience larger costs regarding fruit production and the fraction of undispersed fruits remaining on the plant.

Two fruit traits were significantly selected in our study system: seed dry weight and sugar content, the former being related to seed survival^22^, and the latter to its attractiveness to the disperser in terms of energetic reward^26^. Given the positive and significant correlation between sugar content and crop size, it is possible that mistletoe crop size acts as a signal of the nutritional content for *D. gliroides*, as happens in other plant-frugivore systems^27^,^28^. This result depicts a intriguing scenario, in which seed mass is more relevant at native forest-dominated habitats, where there are less intra- and inter-specific competition (i.e., other fleshy-fruited plants with similar phenologies), but host quality is a limiting factor because the most abundant host species (*Pluchea absinthioides*) experiences high inter-season mortality, compared to early-successional high-quality hosts (e.g., *Aristotelia chilensis, Rhaphithamnus spinosus*) found at the transformed habitat (F.E. Fontúrbel, personal observation). On the other hand, sugar content becomes more relevant in transformed habitats, where sugar content –signalled through crop size– might play a decisive role in attracting seed dispersers that are strongly influenced by a mixed neighbourhood of fruiting species.

Environmental features affected fruit traits under selection in some extent. It is likely that the environment is covarying with plant phenotype and fitness beyond the trait value itself^29^. Taking into account the covariation between environmental variables and phenotypic selection gradients allows for a better understanding of the observed patterns and also allows to determine what portion of the fitness-trait covariance is related to phenotypic selection in a strict sense and what proportion is influenced by environmental features^29^. This issue was solved, at least in part, with the SEM model fitted and our field experiment, which showed causal relationships between seed weight - sugar content variation and the structural effect of canopy complexity and sunlight incidence upon plant fitness. Besides environmental effects the plant traits analysed here may have low heritability and might be influenced by maternal effects (i.e., the genotype of the parent plant may influence the offspring phenotype). The use of molecular markers could provide a valuable insight on those issues^30^ and a better understanding of the potential for evolution of *T. corymbosus* in anthropogenic habitats.

Additionally, frugivore-mediated selection significantly depended on mistletoe’s spatial structure. Spatial arrangement is that much important as environmental features, since plant aggregation influences dispersal distance (and thus, gene flow) and originate a positive-feedback loop driving aggregated plant clusters even more aggregated each generation^31^,^32^. Aggregated plants have greater chances to be visited and its seed dispersed respect to isolated plants, which are unlikely to be visited due to displacements costs and enhanced predation risks^33^. Our study system shows that frugivore-mediated selection in a habitat transformation scenario is a context-dependent phenomenon^34^, in which extreme situations (on the one hand, the original native habitat, and on the other hand, a transformed habitat with a highly aggregated spatial distribution) may be favouring the persistence of a highly specialized plant-disperser system, which is known to be disrupted and even driven locally extinct by habitat fragmentation^35^. This situation illustrates Fahrig, *et al*.^36^ functional landscape heterogeneity definition, showing that this scenario has a neutral -or even somewhat benefic- effect on *T. corymbosus* recruitment and persistence.

Frugivore-mediated selection on *Tristerix corymbosus*, as many mutualistic systems (e.g., refs 37,38), varies according to the ecological context in which it occurs. As we hypothesized, habitat structure and microclimate influenced seed dispersal and phenotypic selection along a habitat transformation gradient, with a complex combination of spatial arrangement and environmental effects, depicting a scenario in which extreme situations are favouring *T. corymbosus* persistence related to *D. gliroides*’ differential response to phenotypic traits of particular relevance at each local context. Human disturbances represent strong sources of evolutionary change^19^, therefore its study in an eco-evolutionary framework is necessary in order to ensure the persistence of native species -and more importantly- of ecological interactions^18^,^39^.

## Methods

### Study site and species

We conducted this study at the Valdivian Coastal Reserve (39°57′S 73°34′W), a private protected area owned and administrated by The Nature Conservancy^40^. It constitutes one of the largest remnants (50,530 ha) of native temperate rainforest in southern South America, which present many endemic species but is currently facing increasing levels of anthropogenic disturbance^41^. This large forest remnant presents a complex habitat mosaic comprising old- and second-growth native stands (which were referred as ‘native habitat’ hereafter), and exotic *Eucalyptus globulus* plantations (12–20 years old) with a dense native understory regenerated in between (which were referred as ‘transformed habitat’ hereafter)^42^. Even when this anthropogenic disturbance is recent, the extent of the habitat modification is likely to trigger quick evolutionary responses^18^,^19^.

Canopy at the native habitat is dominated by *Nothofagus dombeyi, N. pumilio* and *Eucryphia cordifolia*, whereas the exotic *E. globulus* is the only canopy species at the transformed habitat, leaving the understory more exposed to sunlight. Understory vegetation at the native forest is dominated by *Laurelia philippiana, Mitraria coccinea* and *Drimys winteri*, with sparse clusters of the native bamboo *Chusquea quila* and a few vines of *Lapageria rosea*. At the transformed habitat, the shade-intolerant species *Rhaphithamnus spinosus, Aristotelia chilensis, Luma apiculata, Ugni molinae* and *Fuchsia magellanica* dominate understory vegetation, altogether with dense *C. quila* clusters and abundant vines of *L. rosea*.

*Tristerix corymbosus* (Loranthaceae) is an evergreen hemiparasitic mistletoe, which parasitizes about 30 different host plants (including some exotic species), where *Nothofagus dombeyi, Aristotelia chilensis, Rhaphithamnus spinosus* and *Pluchea absinthioides* are the most common hosts at the study area. This mistletoe has single-seeded berries (length 9.56 ± 0.06 mm, width 5.30 ± 0.03 mm), fruiting from December to March (austral summer season). *Tristerix corymbosus* is considered as a keystone species at the South American temperate rainforests, because of its winter-flowering phenology that provides food resources in scarcity periods and sugar-rich fruits during the summer^43^. This mistletoe is dispersed only by two species: the Chilean Mockingbird (*Mimus thenca*) at its northern distribution (30°–37°S), and the arboreal marsupial *Dromiciops gliroides* at its southern distribution (37°–42°S)^44^. Avian dispersal in this mistletoe is apparently precluded by a fruit colour polymorphism at its southern range^44^. Although *T. corymbosus* completely depends on *D. gliroides* for dispersing its seeds, this marsupial has a broad diet including fleshy fruits of at least 16 species^45^, insects, and eggs^46^. In fact, *D. gliroides* cannot sustain a diet based only on fruits or insects^47^.

### Data collection

Between July 2011 and December 2012, we surveyed the study area and located 278 *T. corymbosus* plants parasitizing 197 host trees along the Reserve; no mistletoes were found parasitizing *E. globulus* trees. From those, 70 adult plants were accessible and fruiting during the sampling period (January-March, corresponding to the austral summer when fruits are ripe). We tagged each plant and recorded its location using a Garmin Map 62 s GPS device (≤3 m error). Fourteen of the host plants included in the study presented more than one mistletoe; to avoid pseudoreplication we only sampled one mistletoe per host (to have a common sampling criterion we always selected the largest mistletoe). We studied this plant-frugivore system for two consecutive summer seasons, in 2012 and 2013. We determined each plant’s crop size by counting all the ripe fruits present. After that, we randomly took two samples of 10 fruits each from every plant to record traits and perform germination trials. Also 10 random fruits were marked with a water-based non-toxic paint to quantify fruit removal, painted fruits were counted five days later; seed collectors were placed underneath the sampled plants to account for fallen fruits and avoid counting them as removed. Complementarily, to quantify *D. gliroides* visits we set infrared trap-cameras in front of each sampled mistletoe, operated in video mode for 48 straight hours. Camera-trap monitoring approach has been proven to be effective for assessing *T. corymbosus* – *D. gliroides* interactions (e.g., ref. 42), allowing longer monitoring periods than visual estimations with no researcher interference^48^. All mistletoes were monitored within one week.

Collected fruits were taken to a field-based laboratory, where we measured fruit diameter (using a 0.01 mm precision calliper) and sugar content (using a handheld brix refractometer, precision ± 0.1%). Seeds were dried at room temperature for one week and then weighted using a precision scale (±0.0001 g). A second batch of fruits was used for germination trials. We conducted germination experiments within 24 hours from the collection; pericarp was manually removed and seeds were placed in Petri dishes with wet filter paper, and checked daily for five days (as most seeds germinate within the third day). As *T. corymbosus* has recalcitrant seeds, they germinate immediately without passing through disperser’s gut and they do not need any treatment to activate germination^49^. Therefore, its germination could be studied without passing first through the disperser, with germination rates ≥80%.

As habitat structure varied considerably along the study area^42^, we measured the following environmental variables that might be influencing *T. corymbosus* fruit traits in a 5-m buffer from each plant: shrub cover (visually estimated as percentage), bamboo (*Chusquea quila*) cover (visually estimated as percentage), stem density (the number of DBH ≥ 1 cm stems within the buffer), air temperature (° Celsius), relative humidity (using a handheld thermohygrometer), and luminosity (using a handheld luxometer). To inquire into a potential correlation between habitat variables and fruit traits, we performed a field experiment at the beginning of the fruiting season (early November), when all fruits were unripe. We shaded three mistletoe plants at the transformed habitat, and opened a gap (by trimming vegetation with gardener scissors) on another three plants at the native habitat, from which we had fruit trait measurements (i.e., fruit diameter, seed dry weight and sugar content) from the previous fruiting season. The reduced sample size of this experiment was due to intervention restrictions imposed by the protected area regulations. When fruits were ripe, we collected five fruits per plant, and measured their traits again. The purpose of this experiment was to determine whether fruit traits vary according to sunlight exposure conditions.

### Habitat transformation gradient

As the study area presents a complex and heterogeneous habitat mosaic^42^, assigning each sampled mistletoe to a habitat type was a challenging task. Hence, we used two complementary approaches to overcome this situation. First, we constructed habitat transformation gradient covariates using the 70 sampled plants. For doing so, we used the approach of García and Chacoff^50^, defining three non-overlapping buffer rings from each plant: 0–50 m, 50–100 m, and 100–250 m. At each buffer, we determined the proportion of native habitat present using aerial images, digital cartography and field surveys. These calculations were made using ArcMap 10.1 GIS software (ESRI, Redlands CA). The second approach consisted in splitting sampled plants in two discrete groups regarding the dominant habitat type (native/transformed). We set a threshold of 67% of native habitat (corresponding to the median value of the 0–50 m buffer ring), and plants above this threshold were considered as native habitat, and those below the threshold as transformed habitat.

### Data analysis

For analytic purposes, we defined the effectiveness of the seed disperser vector as the product of *D. gliroides* visit rates (number of visits per hour) and fruit removal rates (percentage of fruits removed). Absolute plant fitness was defined as the product of fruit removal rate x seed germination. First, we correlated raw variables with each other and with habitat covariates by using a non-parametric Spearman correlation test.

Raw trait data (i.e., fruit diameter, seed dry weight, and sugar content), its quadratic (i.e., the squared values) and correlational (i.e., the product of each pairwise trait combination) terms were standardized to mean = 0 and variance = 1 to make them comparable^51^. With those standardized values, we used a modified version of Lande and Arnold^51^ equations to estimate linear and non-linear selection gradients (i.e., the slope of the plant trait and fitness values in the equation, representing the direction and strength of selection); relative plant fitness was estimated as: absolute fitness/mean (absolute fitness) in the population. We made two modifications to the original method: (1) we included crop size as a covariate, and (2) given that *T. corymbosus*, as most mistletoes, has an aggregated spatial distribution, we made this analysis spatially explicit by using Generalized Additive Models (GAM) with a spline term containing UTM coordinates of each sampled plant in order to deal with potential spatial autocorrelation^52^,^53^. In a previous work, we determined that a spatially explicit GAM was the best choice to deal with spatial autocorrelation^42^, therefore we decided to use that approach. Hence, modified equations were expressed as:


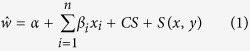



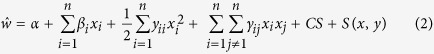


where 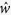 = relative fitness, β_i_ = directional selection gradients, γ_ii_ = stabilizing/disruptive selection gradients, γ_ij_ = correlational selection gradients, CS = crop size (as covariate), S(x, y) = spatially explicit term operated as a spline non-linear function.

Selection differentials (i.e., the expected phenotypic change) were calculated as the covariance between traits and relative fitness values, and its significance was assessed through a Pearson correlation test^54^. We also used the spatially explicit GAM approach to assess the relationship between crop size (log-transformed) and relative fitness; crop size was not assessed as trait as it represents a higher hierarchical level of the plant’s phenotype and it may lead to misleading interpretations as it provides a different kind of signal to frugivores than individual fruits. While crop size influences the forager decision whether to visit or not the plant, individual fruit traits influence consumption patterns and residence time in plants^55^,^56^. Notwithstanding, we included crop size as a covariate in the selection gradient equations. As some data examined had non-normal distributions, we verified each coefficient (except the spatial term) significance level using bootstrapping procedures (with 5,000 iterations)^12^,^14^.

Further, to explore the effects of habitat transformation on the selective forces acting upon *T. corymbosus* dispersal-related traits, we used a Structural Equation Modelling (SEM hereafter) approach^57^,^58^. This procedure allows examining simultaneously the causal relationships of fruit traits and environmental covariates on plant fitness, and allows the examination of the model showing the best fit to the covariance structure of data^59^. We contrasted six competing models (Figure S2), which were assessed using a chi-squared goodness-of-fit test. SEM modelling procedures were conducted in STATA 12.0^60^.

Then, we split up our data in two discrete groups (native and transformed habitat) as described above. We first described raw data using the dichotomous criterion defined above, native and transformed habitat values were compared using a non-parametric Mann-Whitney test. Additionally, to conduct a global comparison between habitat types, we conducted a non-parametric MANOVA test, as model residuals were non-normally distributed. For those traits that had non-significant directional gradients, we estimated the minimum sample required (MSR) for obtaining statistical significance at α = 0.05, which is calculated as MSR = (*t*σ/β)^2^, where *t* was set to 1.96, β is the selection gradient and σ its standard deviation^61^; when MSR exceeded the population size we discarded sample size issues. To compare selection coefficients between habitat types, we performed an analysis of covariance (ANCOVA) using habitat type as factor, plant fitness as response, and fruit traits as covariates^54^; P values were adjusted for multiple comparisons using a Bonferroni sequential adjustment. Aiming to relate trait’s variation to environmental features, we correlated raw trait values with untransformed structural and microclimate features using a Spearman correlation test. Field experiment data was analysed using paired t-tests.

Finally, we constructed a spatially explicit representation of the sampled mistletoes to visualize plant arrangement, superimposing size plots of crop size, sugar content, *Dromiciops gliroides* visit rates, seed disperser effectiveness, plant’s fitness^62^, and a kriging map of the proportion of native forest along the study area. This approach allows a visual interpretation of the actual spatial configuration and the spatial variation of determinant steps of plant recruitment. Values are presented as mean ± 1 SE. All statistical procedures were conducted in R 3.1.0^63^ and external packages (boot, car, deldir, ggplot2, mgcv, spdep, spatstat, vegan).

### Data accesibility

Original data may be accessed at: http://dx.doi.org/10.6084/m9.figshare.4614769.

## Additional Information

**How to cite this article:** Fontúrbel, F. E. and Medel, R. Frugivore-Mediated Selection in A Habitat Transformation Scenario. *Sci. Rep.* **7**, 45371; doi: 10.1038/srep45371 (2017).

**Publisher's note:** Springer Nature remains neutral with regard to jurisdictional claims in published maps and institutional affiliations.

## Supplementary Material

Supplementary Information

## Figures and Tables

**Table 1:** Frugivore-mediated selection coefficients for 70 *Tristerix corymbosus* plants along a habitat disturbance gradient.

Plant trait	β′	S_i_	γ_ii_′	S_j_
Fruit diameter	0.021 (0.033)^NS^	0.012	0.024 (0.033)^NS^	0.014^*^
Seed weight	0.088 (0.033)^*^	−0.058	0.076 (0.033)^*^	0.046
Sugar content	−0.066 (0.029)^*^	−0.078^*^	−0.074 (0.029)^*^	−0.075^*^
Fruit diameter x seed weight	—	—	0.156 (0.061)^*^	—
Fruit diameter x sugar content	—	—	−0.037 (0.083)^NS^	—
Seed weight x sugar content	—	—	−0.056 (0.083)^NS^	—

Directional (β′) and stabilizing/disruptive (γ_ii_′) standardized selection coefficients are presented (standard errors are shown in parentheses). Selection differentials are presented for directional (S_i_) and stabilizing/disruptive (S_j_) gradients. Significance of each gradient was tested using a bootstrapping procedure; ^NS^ = not significant (confidence interval overlapped zero), *Significant (confidence interval do not overlapped zero).

**Table 2:** Analysis of covariance (ANCOVA) of trait effect on plant’s fitness between native and transformed habitats.

Source	df	SS	F
Habitat (H)	1	0.392	7.134^**^
Fruit diameter (F)	1	0.012	0.201
Seed dry weight (W)	1	0.175	0.175
Sugar content (S)	1	0.607	11.055^**a^
H × F	1	0.094	1.715
H × W	1	0.167	3.049
H × S	1	0.247	4.497^*^
Error	62	3.405	

Degrees of freedom (df), sum of squares (SS), and F values are presented. Significance codes: *P < 0.05, **P < 0.01, ^a^coefficients that retained significance after sequential Bonferroni adjustment.

## References

[b1] Sapir Y. & Armbruster W. S. Pollinator-mediated selection and floral evolution: from pollination ecology to macroecology. New Phytol. 188, 303–306, doi: 10.1111/j.1469-8137.2010.03467.x (2010). 20941844

[b2] Lomáscolo S. B., Levey D. J., Kimball R. T., Bolker B. M. & Alborn H. T. Dispersers shape fruit diversity in *Ficus* (Moraceae). P. Natl. Acad. Sci. USA 107, 14668–14672, doi: 10.1073/pnas.1008773107 (2010). PMC2930445 20679219

[b3] Lomáscolo S. B., Speranza P. & Kimball R. T. Correlated evolution of fig size and color supports the dispersal syndromes hypothesis. Oecologia 156, 783–796, doi: 10.1007/s00442-008-1023-0 (2008). 18386067

[b4] Chapin F. S. III . Consequences of changing biodiversity. Nature 405, 234–242, doi: 10.1038/35012241 (2000). 10821284

[b5] Kolb A. & Diekmann M. Effects of life-history traits on responses of plant species to forest fragmentation. Conserv. Biol. 19, 929–938, doi: 10.1111/j.1523-1739.2005.00065.x (2005).

[b6] McConkey K. R. . Seed dispersal in changing landscapes. Biol. Conserv. 146, 1–13, doi: 10.1016/j.biocon.2011.09.018 (2012).

[b7] Schleuning M. . Forest fragmentation and selective logging have inconsistent effects on multiple animal-mediated ecosystem processes in a tropical forest. PLoS ONE 6, e27785, doi: 10.1371/journal.pone.0027785 (2011). 22114695 PMC3218041

[b8] Ewers R. M. & Didham R. K. Confounding factors in the detection of species responses to habitat fragmentation. Biol. Rev. 81, 117–142, doi: 10.1017/S1464793105006949 (2006). 16318651

[b9] Moran C. & Catterall C. P. Responses of seed-dispersing birds to amount of rainforest in the landscape around fragments. Conserv. Biol. 28, 551–560, doi: 10.1111/cobi.12236 (2014). 24548306

[b10] Caley M. J., Buckley K. A. & Jones G. P. Separating ecological effects of habitat fragmentation, degradation, and loss on coral commensals. Ecology 82, 3435–3448, doi: 10.2307/2680163 (2001).

[b11] Melo F. P. L., Arroyo-Rodríguez V., Fahrig L., Martínez-Ramos M. & Tabarelli M. On the hope for biodiversity-friendly tropical landscapes. Trends Ecol. Evol. 28, 462–468, doi: 10.1016/j.tree.2013.01.001 (2013). 23375444

[b12] Jordano P. Frugivore-mediated selection on fruit and seed size: birds and St. Lucie’s cherry, *Prunus mahaleb*. Ecology 76, 2627–2639, doi: 10.2307/2265833 (1995).

[b13] Thorpe A. S., Aschehoug E. T., Atwater D. Z. & Callaway R. M. Interactions among plants and evolution. J. Ecol. 99, 729–740, doi: 10.1111/j.1365-2745.2011.01802.x (2011).

[b14] Weber A. & Kolb A. Population size, pollination and phenotypic trait selection in *Phyteuma spicatum*. Acta Oecol. 47, 46–51, doi: 10.1016/j.actao.2012.12.004 (2013).

[b15] García C., Jordano P., Arroyo J. M. & Godoy J. A. Maternal genetic correlations in the seed rain: effects of frugivore activity in heterogeneous landscapes. J. Ecol. 97, 1424–1435, doi: 10.1111/j.1365-2745.2009.01577.x (2009).

[b16] Carvalho C. S., Galetti M., Colevatti R. G. & Jordano P. Defaunation leads to microevolutionary changes in a tropical palm. Sci Rep-UK 6, doi: 10.1038/srep31957 (2016). 10.1038/srep31957 PMC4989191 27535709

[b17] Perez-Mendez N., Jordano P., Garcia C. & Valido A. The signatures of Anthropocene defaunation: cascading effects of the seed dispersal collapse. Sci Rep-UK 6, doi: 10.1038/srep24820 (2016). 10.1038/srep24820 PMC4835773 27091677

[b18] Stockwell C. A., Hendry A. P. & Kinnison M. T. Contemporary evolution meets conservation biology. Trends Ecol. Evol. 18, 94–101, doi: 10.1016/S0169-5347(02)00044-7 (2003).

[b19] Kinnison M. T. & Hairston N. G. Eco-evolutionary conservation biology: contemporary evolution and the dynamics of persistence. Funct. Ecol. 21, 444–454, doi: 10.1111/j.1365-2435.2007.01278.x (2007).

[b20] Herrera J. M., Morales J. M. & García D. Differential effects of fruit availability and habitat cover for frugivore-mediated seed dispersal in a heterogeneous landscape. J. Ecol. 99, 1100–1107, doi: 10.1111/j.1365-2745.2011.01861.x (2011).

[b21] García D., Martínez D., Herrera J. M. & Morales J. M. Functional heterogeneity in a plant-frugivore assemblage enhances seed dispersal resilience to habitat loss. Ecography 36, 197–208, doi: 10.1111/j.1600-0587.2012.07519.x (2013).

[b22] Chacón P. & Bustamante R. O. The effects of seed size and pericarp on seedling recruitment and biomass in *Cryptocarya alba* (Lauraceae) under two contrasting moisture regimes. Plant Ecol. 152, 137–144, doi: 10.1023/A:1011463127918 (2001).

[b23] Alcantara J. M. & Rey P. J. Conflicting selection pressures on seed size: evolutionary ecology of fruit size in a bird-dispersed tree, Olea europaea. J. Evolution. Biol. 16, 1168–1176, doi: 10.1046/j.1420-9101.2003.00618.x (2003). 14640408

[b24] Sobral M., Larrinaga A. R. & Guitian J. Do seed-dispersing birds exert selection on optimal plant trait combinations? Correlated phenotypic selection on the fruit and seed size of hawthorn (*Crataegus monogyna*). Evol. Ecol. 24, 1277–1290, doi: 10.1007/s10682-010-9380-7 (2010).

[b25] Palacio F. X., Girini J. M. & Ordano M. Linking the hierarchical decision-making process of fruit choice and the phenotypic selection strength on fruit traits by birds. J. Plant Ecol., doi: 10.1093/jpe/rtw063 (2016).

[b26] Izhaki I. In Seed Dispersal and Frugivory: Ecology, Evolution, and Conservation (eds Levey D. J., Silva W. R. & Galetti M.) Ch. 11, 161–176 (CAB International, 2002).

[b27] Cazetta E., Galetti M., Rezende E. L. & Schaefer H. M. On the reliability of visual communication in vertebrate-dispersed fruits. J. Ecol. 100, 277–286, doi: 10.1111/j.1365-2745.2011.01901.x (2012).

[b28] Schaefer H. M., Schaefer V. & Levey D. J. How plant-animal interactions signal new insights in communication. Trends Ecol. Evol. 19, 577–584, doi: 10.1016/j.tree.2004.08.003 (2004).

[b29] MacColl A. D. C. The ecological causes of evolution. Trends Ecol. Evol. 26, 514–522, doi: 10.1016/j.tree.2011.06.009 (2011). 21763030

[b30] Castellanos M. C., Alcantara J. M., Rey P. J. & Bastida J. M. Intra-population comparison of vegetative and floral trait heritabilities estimated from molecular markers in wild *Aquilegia* populations. Mol. Ecol. 20, 3513–3524, doi: 10.1111/j.1365-294X.2011.05094.x (2011). 21504491

[b31] Carlo T. A. & Morales J. M. Inequalities in fruit-removal and seed dispersal: consequences of bird behaviour, neighbourhood density and landscape aggregation. J. Ecol. 96, 609–618, doi: 10.1111/j.1365-2745.2008.01379.x (2008).

[b32] Morales J. M. & Carlo T. A. The effects of plant distribution and frugivore density on the scale and shape of dispersal kernels. Ecology 87, 1489–1496, doi: 10.1890/0012-9658(2006)87[1489:TEOPDA]2.0.CO;2 (2006). 16869425

[b33] Fedriani J. M., Wiegand T. & Delibes M. Spatial pattern of adult trees and the mammal-generated seed rain in the Iberian pear. Ecography 33, 545–555, doi: 10.1111/j.1600-0587.2009.06052.x (2010).

[b34] Maron J. L., Baer K. C. & Angert A. L. Disentangling the drivers of context-dependent plant–animal interactions. J. Ecol. 102, 1485–1496, doi: 10.1111/1365-2745.12305 (2014).

[b35] Rodríguez-Cabal M. A., Aizen M. A. & Novaro A. J. Habitat fragmentation disrupts a plant-disperser mutualism in the temperate forest of South America. Biol. Conserv. 139, 195–202, doi: 10.1016/j.biocon.2007.06.014 (2007).

[b36] Fahrig L. . Functional landscape heterogeneity and animal biodiversity in agricultural landscapes. Ecol. Lett. 14, 101–112, doi: 10.1111/j.1461-0248.2010.01559.x (2011). 21087380

[b37] Cuartas-Hernández S., Núñez-Farfán J. & Smouse P. E. Restricted pollen flow of *Dieffenbachia seguine* populations in fragmented and continuous tropical forest. Heredity 105, 197–204, doi: 10.1038/hdy.2009.179 (2010). 20029453

[b38] Prasad S. & Sukumar R. Context-dependency of a complex fruit-frugivore mutualism: temporal variation in crop size and neighborhood effects. Oikos 119, 514–523, doi: 10.1111/j.1600-0706.2009.17971.x (2010).

[b39] Kinnison M. T., Hendry A. P. & Stockwell C. A. Contemporary evolution meets conservation biology II: Impediments to integration and application. Ecol. Res. 22, 947–954, doi: 10.1007/s11284-007-0416-6 (2007).

[b40] Delgado C. Plan de manejo Reserva Costera Valdiviana [Management plan of the Valdivian Coastal Reserve]. (The Nature Conservancy, 2010).

[b41] Mittermier R. A. . Hotspots revisited: Earth’s biologically richest and most threatened terrestrial regions. (CEMEX, 2005).

[b42] Fontúrbel F. E., Jordano P. & Medel R. Scale-dependent responses of pollination and seed dispersal mutualisms in a habitat transformation scenario. J. Ecol. 103, 1334–1343, doi: 10.1111/1365-2745.12443 (2015).

[b43] Aizen M. A. Influences of animal pollination and seed dispersal on winter flowering in a temperate mistletoe. Ecology 84, 2613–2627, doi: 10.1890/02-0521 (2003).

[b44] Amico G. C., Rodríguez-Cabal M. A. & Aizen M. A. Geographic variation in fruit colour is associated with contrasting seed disperser assemblages in a south-Andean mistletoe. Ecography 34, 318–326, doi: 10.1111/j.1600-0587.2010.06459.x (2011).

[b45] Amico G. C., Rodríguez-Cabal M. A. & Aizen M. A. The potential key seed-dispersing role of the arboreal marsupial *Dromiciops gliroides*. Acta Oecol. 35, 8–13, doi: 10.1016/j.actao.2008.07.003 (2009).

[b46] Fontúrbel F. E., Franco M., Rodríguez-Cabal M. A., Rivarola M. D. & Amico G. C. Ecological consistency across space: a synthesis of the ecological aspects of *Dromiciops gliroides* in Argentina and Chile. Naturwissenschaften 99, 873–881, doi: 10.1007/s00114-012-0969-2 (2012). 22996392

[b47] Cortés P. A., Franco M., Sabat P., Quijano S. A. & Nespolo R. F. Bioenergetics and intestinal phenotypic flexibility in the microbiotherid marsupial (*Dromiciops gliroides*) from the temperate forest in South America. Comp. Biochem. Physiol. A 160, 117–124, doi: 10.1016/j.cbpa.2011.05.014 (2011). 21627996

[b48] Fontúrbel F. E., Candia A. B. & Botto-Mahan C. Nocturnal activity patterns of the monito del monte (*Dromiciops gliroides*) in native and exotic habitats. J. Mammal. 95, 1199–1206, doi: 10.1644/13-MAMM-A-304 (2014).

[b49] Gonzalez W. L., Suarez L. H. & Medel R. Outcrossing increases infection success in the holoparasitic mistletoe *Tristerix aphyllus* (Loranthaceae). Evol. Ecol. 21, 173–183, doi: 10.1007/s10682-006-0021-0 (2007).

[b50] García D. & Chacoff N. P. Scale-dependent effects of habitat fragmentation on hawthorn pollination, frugivory, and seed predation. Conserv. Biol. 21, 400–411, doi: 10.1111/j.1523-1739.2006.00593.x (2007). 17391190

[b51] Lande R. & Arnold S. The measurement of selection on correlated characters. Evolution 37, 1210–1226, doi: 10.2307/2408842 (1983). 28556011

[b52] Dormann C. F. . Methods to account for spatial autocorrelation in the analysis of species distributional data: a review. Ecography 30, 609–628, doi: 10.1111/j.2007.0906-7590.05171.x (2007).

[b53] Marrot P., Garant D. & Charmantier A. Spatial autocorrelation in fitness affects the estimation of natural selection in the wild. Methods Ecol. Evol. 6, 1474–1483, doi: 10.1111/2041-210x.12448 (2015).

[b54] Murúa M., Espinoza C., Bustamante R., Marin V. H. & Medel R. Does human-induced habitat transformation modify pollinator-mediated selection? A case study in *Viola portalesia* (Violaceae). Oecologia 163, 153–162, doi: 10.1007/s00442-010-1587-3 (2010). 20213152

[b55] Ortiz-Pulido R. & Rico-Gray V. The effect of spatio-temporal variation in understanding the fruit crop size hypothesis. Oikos 91, 523–527, doi: 10.1034/j.1600-0706.2000.910314.x (2000).

[b56] Ortiz-Pulido R., Albores-Barajas Y. V. & Diaz S. A. Fruit removal efficiency and success: influence of crop size in a neotropical treelet. Plant Ecol. 189, 147–154, doi: 10.1007/s11258-006-9175-7 (2007).

[b57] Rey P. J. . The geographic mosaic in predispersal interactions and selection on *Helleborus foetidus* (Ranunculaceae). J. Evolution. Biol. 19, 21–34, doi: 10.1111/j.1420-9101.2005.00992.x (2006). 16405573

[b58] Grace J. B. Structural equation modeling and natural systems. (Cambridge University Press, 2006).

[b59] Mitchell R. J. Testing evolutionary and ecological hypotheses using path-analysis and structural equation modeling. Funct. Ecol. 6, 123–129, doi: 10.2307/2389745 (1992).

[b60] Stata Statistical Software: Release 12 (StataCorp LP, College Station, TX, 2011).

[b61] Medel R. Assessment of parasite-mediated selection in a host-parasite system in plants. Ecology 81, 1554–1564, doi: 10.1890/0012-9658(2000)081[1554:AOPMSI]2.0.CO;2 (2000).

[b62] Bivand R. S., Pebesma E. J. & Gómez-Rubio V. Applied spatial data analysis with R. (Springer, 2008).

[b63] R: A language and environment for statistical computing, reference index version 3.1.0. (Foundation for Statistical Computing, Viena, Austria, 2014).

