# Supplementary material for: Frugivore-Mediated Selection in A Habitat Transformation Scenario
Source: Sci Rep. 2017 Mar 28;7:45371. doi: 10.1038/srep45371 (PMC5368566; doi:10.1038/srep45371)
Supplement: Supplementary Information [file srep45371-s1.doc]

**SUPPLEMENTARY INFORMATION**

**Frugivore-mediated selection in a habitat transformation scenario**

Francisco E. Fontúrbel & Rodrigo Medel

Table S1. Spearman correlation coefficients between plant characters (crop size, fruit diameter, seed dry weight, and sugar contents), fecundity components (fruit removal, seed disperser effectiveness (SDE), and seed germination), and the proportion of native habitat (NH) at three progressive spatial scales. Significance levels: * P < 0.05, ** P < 0.01.

| Character | Crop size | Fruit diam | Seed wgt | Sugar % | Removal | SDE | Germ |
| --- | --- | --- | --- | --- | --- | --- | --- |
| Fruit diam | 0.116 |  |  |  |  |  |  |
| Seed wgt | 0.258* | 0.628** |  |  |  |  |  |
| Sugar % | 0.514** | -0.003 | 0.225 |  |  |  |  |
| Removal | -0.442** | 0.056 | 0.153 | -0.147 |  |  |  |
| SDE | -0.282* | -0.143 | -0.067 | -0.143 | 0.581** |  |  |
| Germ | 0.093 | 0.277* | 0.488** | -0.050 | 0.100 | -0.065 |  |
| NH 0-50m | 0.098 | 0.063 | -0.158 | -0.097 | -0,161 | 0.039 | -0.135 |
| NH 50-100m | 0.056 | -0.142 | -0.252* | -0.076 | -0.243* | 0.094 | -0.140 |
| NH 100-250m | 0.056 | -0.073 | -0.293* | -0.092 | -0.264* | 0.071 | -0.104 |

Table S2. Structural equation modeling coefficients, standard errors (SE), z-values, P-values, and 95% confidence intervals for (a) structural, (b) variance and (c) covariance terms. Variables included: light incidence (light), sugar content (sugar), fruit size (fruit), seed dry weight (seed), fruit removal (removal), seed germination (germ), plant fitness (w). LR test of model vs. saturated: χ^2^_11_ = 12.75, P = 0.3098.

|  | Coefficient | SE | z | P | 95% CI low | 95% CI up |
| --- | --- | --- | --- | --- | --- | --- |
| (a) Structural | | | | | | |
| sugar 🡨 light | 0.190 | 0.117 | 1.62 | 0.106 | -0.040 | 0.420 |
| fruit 🡨 light | 0.120 | 0.119 | 1.01 | 0.313 | -0.114 | 0.355 |
| seed 🡨 light | 0.285 | 0.115 | 2.49 | 0.013 | 0.061 | 0.510 |
| removal 🡨 sugar | -0.248 | 0.118 | -2.11 | 0.035 | -0.478 | -0.017 |
| removal 🡨 fruit | -0.130 | 0.118 | -1.11 | 0.268 | -0.361 | 0.100 |
| germ 🡨 seed | 0.439 | 0.107 | 4.11 | < 0.001 | 0.230 | 0.648 |
| w 🡨 removal | 0.263 | 0.008 | 31.61 | < 0.001 | 0.247 | 0.279 |
| w 🡨 germ | 0.086 | 0.008 | 10.33 | < 0.001 | 0.070 | 0.102 |
| (b) Variance | | | | | | |
| ξ sugar | 0.950 | 0.161 |  |  | 0.682 | 1.323 |
| ξ fruit | 0.984 | 0.165 |  |  | 0.708 | 1.368 |
| ξ seed | 0.905 | 0.153 |  |  | 0.650 | 1.261 |
| ξ removal | 0.918 | 0.155 |  |  | 0.659 | 1.279 |
| ξ germ | 0.796 | 0.135 |  |  | 0.571 | 1.108 |
| ξ w | 0.005 | 0.001 |  |  | 0.003 | 0.007 |
| (c) Covariance | | | | | | |
| ξ sugar-fruit | -0.201 | 0.097 | -2.08 | -0.038 | -0.391 | -0.011 |
| ξ fruit-seed | 0.555 | 0.129 | 4.30 | < 0.001 | 0.302 | 0.807 |

Table S3. Between-habitat comparisons for plant (crop size, fruit diameter, seed dry mass, and sugar content) and fecundity (*Dromiciops gliroides* visits per plant, fruit removal, germination, and relative fitness) characteristics. Differences were assessed through non-parametric Mann-Whitney tests. Significance levels: ** P < 0.01

| Variable | Habitat | | Mann-Whitney P-value |
| --- | --- | --- | --- |
|  | Native | Transformed |  |
| Crop size | 54.46 ± 7.98 | 44.63 ± 6.71 | 0.284 |
| Fruit diameter (mm) | 5.29 ± 0.08 | 5.12 ± 0.10 | 0.385 |
| Seed dry mass (mg) | 24.41 ± 1.66 | 26.66 ± 1.82 | 0.356 |
| Sugar content (Brix %) | 17.10 ± 0.91 | 17.52 ± 0.89 | 0.496 |
| *D. gliroides* visits | 1.17 ± 0.39 | 1.20 ± 0.35 | 0.937 |
| Fruit removal (%) | 24.85 ± 4.18 | 50.81 ± 6.17 | 0.003** |
| Germination (%) | 74.91 ± 4.55 | 79.39 ± 4.26 | 0.484 |
| Relative fitness | 0.19 ± 0.04 | 0.41 ± 0.05 | 0.002** |

Table S4. Spearman correlation coefficients between *T. corymbosus* fruit traits and environmental structural and microclimate features. Significance levels: * P < 0.05, ** P < 0.01.

| Trait | Shrub cover | Bamboo cover | Stem density | Air temperature | Relative humidity | Luminosity |
| --- | --- | --- | --- | --- | --- | --- |
| Fruit diameter | -0.078 | 0.289* | -0.138 | 0.098 | -0.199 | 0.176 |
| Seed weight | -0.142 | 0.195 | -0.074 | -0.179 | 0.104 | 0.238* |
| Sugar content | -0.271* | 0.006 | -0.020 | -0.155 | -0.019 | 0.339** |


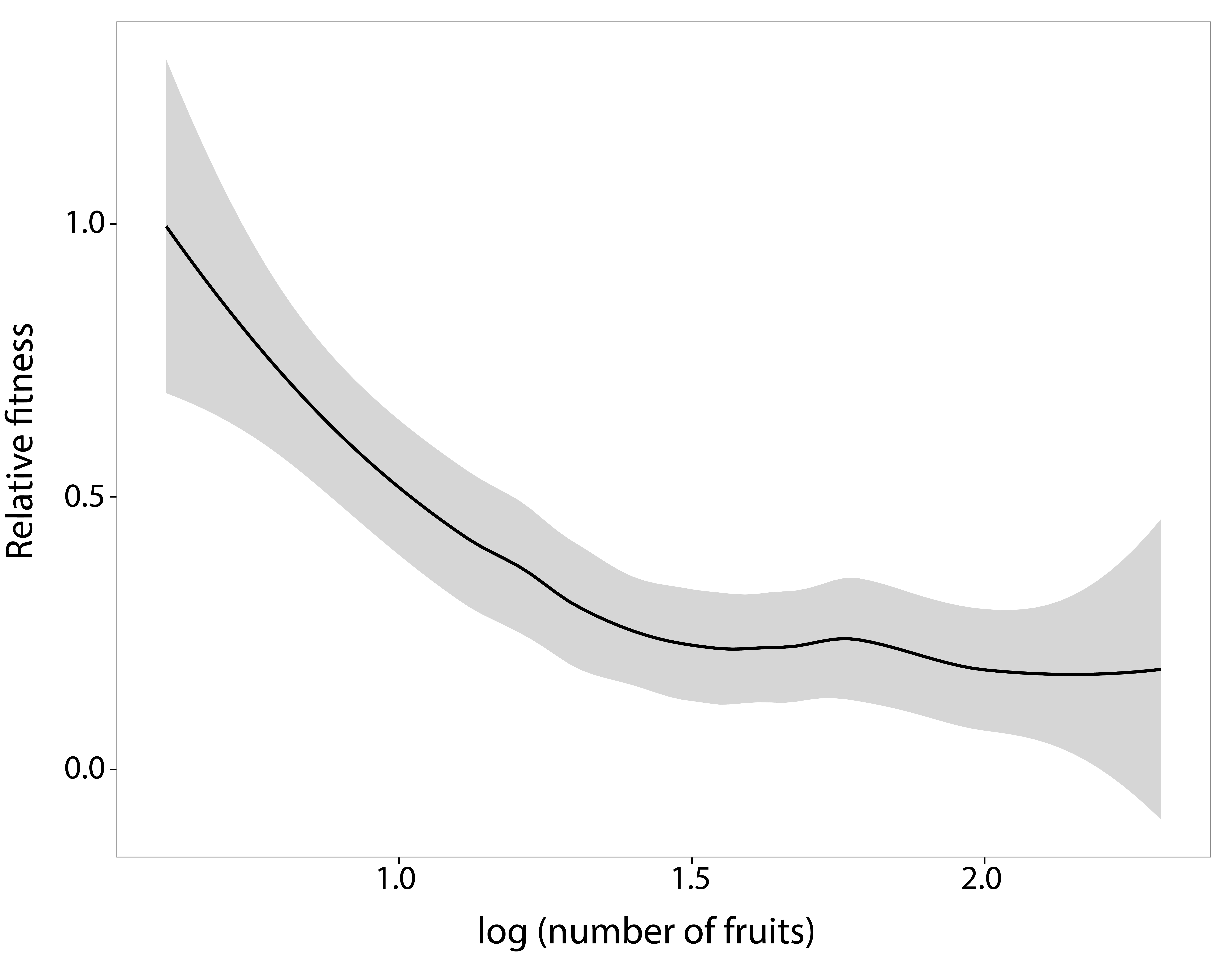


Figure S1. Crop size effect on plant’s relative fitness (spatially explicit GAM, Estimate = -0.31 ± 0.07, P < 0.001; spline (X,Y), F = 3.72, P = 0.002). Relative fitness decreased with crop size up to 40 fruits, and beyond that value the increase in the number of fruits has no visible effects on plant’s relative fitness. Shaded areas represent 95% confidence intervals. Trend and confidence intervals we based on the GAM results (based on loess, depicted using ggplot2 package in R 2.15, parameter settings: size = 1, span = 1).


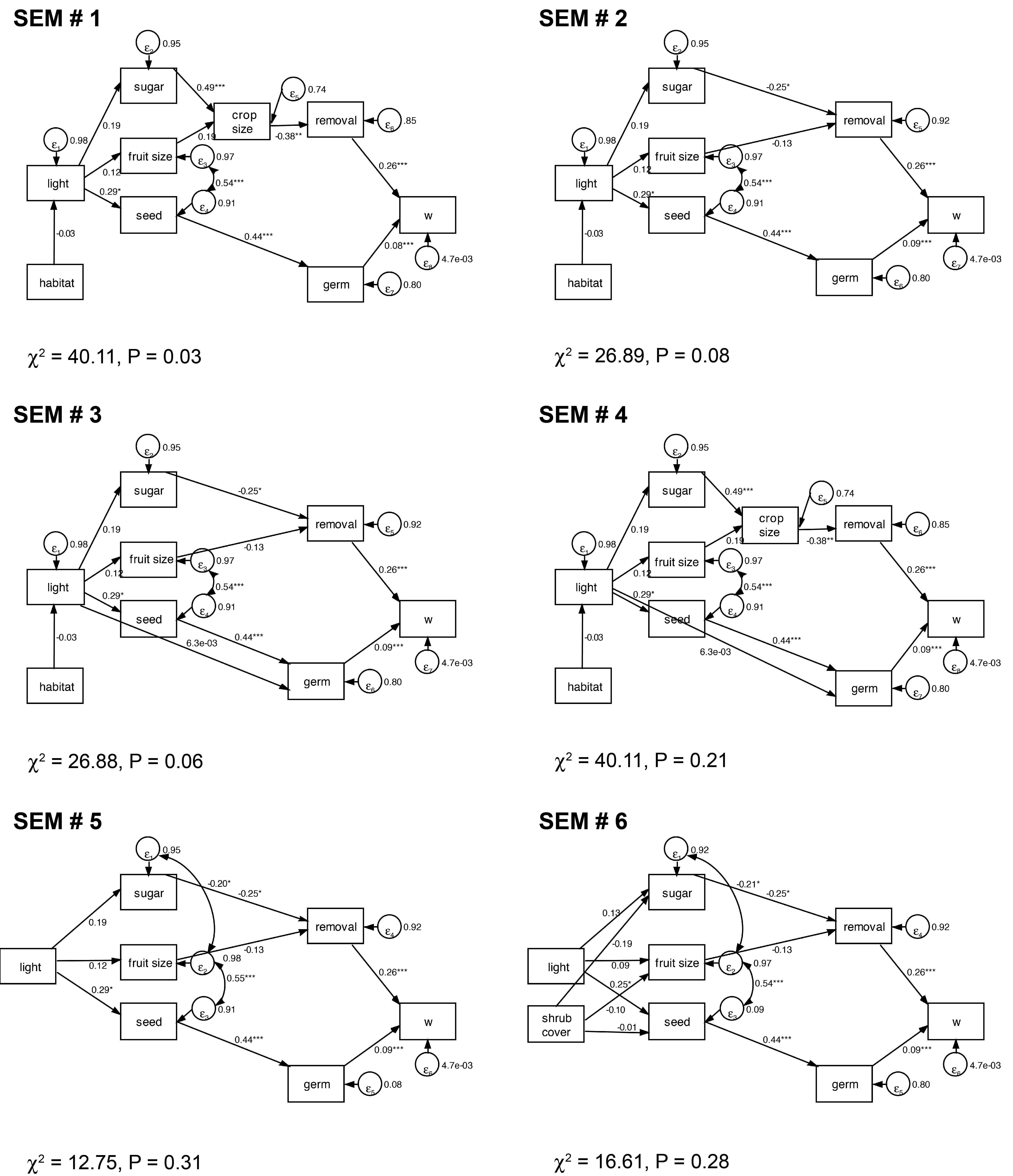


Figure S2. Competing models used for Structural Equation Modelling. Path significance: * P < 0.05, ** P < 0.01, *** P < 0.001. Goodness-of-fit chi-squared test is provided for each competing model.
